# To be continued: family planning continuation among the urban poor in Senegal, a prospective, longitudinal descriptive study.

**DOI:** 10.12688/gatesopenres.12880.1

**Published:** 2018-12-03

**Authors:** Jill Peterson, Aurélie Brunie, Salif Ndeye, Elisabeth Diatta, John Stanback, Dawn Chin-Quee

**Affiliations:** 1 FHI 360, Washington, DC, USA; 2 Centre de Recherche pour le Développement Humain, Sénégal, Dakar, Senegal; 3 IntraHealth International, Dakar, Senegal; 4 FHI 360, Durham, NC, USA

**Keywords:** family planning, continuation, Senegal, urban, FHI 360

## Abstract

**Background: **Given the role that continued use of family planning (FP) by current users plays in increasing contraceptive prevalence rates (CPR), this research aims to measure method-specific continuation rates for fixed-site and community-based program interventions and to document reasons for discontinuation.

**Methods: **This research compared discontinuation rates for clients initiating family planning through two types of strategies—services provided at existing health centers that provided regular, ongoing services, and “one-off” outreach services in communities.  Data collectors surveyed consenting clients who were initiating a modern method, or reinitiating after a break of at least six months, and conducted a follow up survey after seven months.

**Results: **Long acting reversible contraception (LARC) was more commonly initiated through outreach strategies than through fixed sites. LARC made up 65% of methods initiated through the outreach setting and 47% of those initiated through a fixed-site strategy. Continuation rates varied from 99% for intrauterine devices (IUDs) to 77% for injectables and were very similar between outreach and fixed-site strategies, with the exception of oral contraceptive pills (OCPs). Only 65% of outreach initiators continued using OCPs, compared to 84% of fixed-site initiators. Top reported reasons for discontinuation were side effects and little or no sexual relations.

**Conclusions: **Project interventions allowed most women to continue with their chosen method of FP for the seven-month duration of the study whether initiated through fixed site or outreach strategies, showing promise in helping Senegal to increase its CPR. It is feasible to offer both LARC and short acting methods through outreach strategies.  Further research into the sensitivity of demand to the price charged is needed.

## Introduction

Current modern family planning (FP) use among married women in Senegal increased from 10% in 2005 to 23% in 2015 ^1^. This doubling in prevalence could be attributed to several factors, including greater policy and program support for FP by the public sector, fewer stock-outs of contraceptive commodities, strides in community-based provision of oral and injectable contraceptives, and outreach to increase demand for and provision of long-term methods. Despite this encouraging increase in contraceptive prevalence rate (CPR), however, current use remains outpaced by unmet need for FP, which stands at 25% among currently married women ^1^. Furthermore, while urban women are more likely to use modern contraceptive methods than their rural counterparts (33% vs. 17%) ^1^, only 17% of the urban poor (the lowest wealth quintile) use modern methods, compared to 29% in the highest quintile ^2^.

Senegal’s National Family Planning Action Plan (2012–2015) ^3^ noted several challenges to FP use, including potential users’ fear of side effects and of negative health impacts, as well as strong social taboos against FP. The plan also noted challenges to effective FP service delivery, including poor service quality and too few service delivery points. These barriers can affect not only initiation of a method of FP, but also continued use over time—both of which are key components in increasing the CPR. Past research has shown that discontinuation of a method for reasons other than a desire to become pregnant plays a role in unwanted fertility, demonstrating that programs should pay close attention to *current* users of FP as a way to decrease unwanted pregnancies ^4^. Although first-year contraceptive discontinuation rates are improving in Senegal, 2014 Demographic and Health Survey (DHS) ^5^ estimates remain high at 38% for oral contraceptive pills (OCPs) and 41% for injectables. Alternatively, long acting reversible contraception (LARC), including intrauterine devices (IUDs) and implants, has a very low rate of first year discontinuation—less than 7% for implants. Though not included in the DHS, discontinuation rates for IUDS are comparable, or even lower, than those of implants ^6^.

## Background

This research examines continuation of family planning use for clients initiating a FP method through Senegal’s Urban Reproductive Health Initiative, funded by the Bill & Melinda Gates Foundation. Aligned with the Ministry of Health’s efforts to achieve Millennium Development Goals 4 and 5 to improve maternal and child health ^7^, the ISSU ( *Initiative Sénégalaise de Santé Urbaine*) project aimed to both increase the CPR and improve FP service quality specifically for the underserved or urban poor in seven cities: Dakar and its environs, Guédiawaye, Pikine, Mbao, Keur Massar, Mbour, and Kaolack. IntraHealth International coordinated the six-year (2010–2015) ISSU project, working with a consortium of public and private sector partners that implemented demand creation activities and provision of FP through a variety of project strategies.

The present study was designed by FHI 360, the operations research partner under ISSU, and implemented by the *Centre de Recherche pour le Développement Humain* (CRDH). The main objectives were to measure method-specific continuation rates and to document reasons for discontinuation. For scale-up purposes researchers wanted to determine if continuation rates varied depending on the specific service delivery strategy under which the method was first received, and whether clients who initiated a method through an outreach strategy were able to find a permanent source for resupply. Table 1 shows the six ISSU FP delivery strategies included in the study, including two fixed-site and four outreach strategies. Methods and the consultation were free when offered through outreach strategies, whereas through fixed-site strategies, clients paid for their methods and the consultation.

**Table 1.** Program strategy descriptions.

Strategy	Description of activities	District
**Fixed-Site Strategy**		
Midwife approach ( *Approche* *Sages-femmes*)	Increased the midwives available at health facilities to provide FP and MCH services. 20 midwives recruited.	Guédiawaye, Pikine
MSI Social Franchise	Recruited and trained private service delivery providers at “Blue Star” branded clinics to provide full FP/RH services to women seeking services through the private sector.	Keur Massar, Mbao, Pikine
**Outreach Strategy**		
ANSFES ( *Association Nationale* *des Sages-Femmes d’Etat du* *Sénégal*) Outreach Strategy	Implemented reproductive health service delivery by sending mid-wife members of ANSFES into communities to refer women seeking FP to local service delivery points.	Dakar Sud, Dakar Centre, Dakar Ouest, Dakar Nord, Guédiawaye, Kaolack, Keur Massar, Mbao, Mbour, Pikine
Enda Santé Mobile Clinic	Deployed a mobile clinic into underserved areas to offer FP and other services to women of reproductive age.	Keur Massar, Mbao, Mbour
Free municipal community fairs ( *Consultations Foraines* *Municipales Gratuites*) (CFMG)	Organized gatherings of 100–300 community members to learn more about FP options. Same-day provision of methods available for interested women.	Dakar Sud, Dakar Centre, Dakar Ouest, Dakar Nord, Guédiawaye, Kaolack, Keur Massar, Mbao, Mbour, Pikine
Outreach by MSI (Marie Stopes International)	Deployed teams of service delivery staff, including midwives, to provide FP services to women of reproductive age with FP services in underserved areas. Services provided through health posts or huts, or under tents when other options were not available.	Dakar Sud, Dakar Centre, Dakar Ouest, Dakar Nord, Guédiawaye, Keur Massar, Mbao

## Methods

This was a prospective, longitudinal descriptive study in 10 urban districts of Senegal. Data were collected through a survey of women initiating their method through one of the ISSU FP delivery strategies with a follow-up phone survey conducted with the same women after seven months (See Extended data ^8^). During a one-month period, providers screened clients for eligibility—all consenting women aged 18–49 who initiated OCPs, injectables, implants, or IUDs or re-initiated any of these methods after a break of at least six months through one of the six FP delivery strategies, and who were reachable by phone. Providers referred eligible clients to data collectors for an exit interview. We estimated one month of screening would give us a sufficient number of respondents to calculate disaggregated rates by method and strategy. At endline, interviewers made up to five attempts to contact women at varying days and times at the number provided at the time of the initial interview.

Baseline data were collected in March 2015, and endline data in October of that year; both surveys were conducted by trained female interviewers in French or the local language if preferred by the client. Written consent was obtained at baseline for both surveys, with confirmation via oral consent for the phone survey. No compensation was provided. The study was approved by Senegal’s *Comité National d'Ethique pour la Recherche en Santé* (SEN 14/62) and FHI 360’s Protection of Human Subjects Committee (641346-1).

### Data Analysis

Descriptive analyses were conducted in SPSS version 17.0. Baseline and endline data were linked through unique identifiers. Analyses were stratified by fixed-site versus outreach strategy, as appropriate. To examine possible bias that may result from differential attrition, we used descriptive analysis to compare the proportion of those initially enrolled through outreach with those initially enrolled through fixed-site strategies between baseline and endline, as well as the mix of methods initiated at baseline, to ensure the method mix was proportionally similar at endline.

The method-specific continuation rate was defined as the proportion of clients who were still using the same modern method obtained at baseline after seven months. When calculating overall continuation rates, we included women who 1) were still using the same modern method, 2) had switched to another one of the four modern methods without a lapse in coverage of more than 28 days, or 3) had interrupted and subsequently restarted use of the same modern method without a lapse of coverage of 28 days or more. Overall discontinuation was defined as ceasing the use of the modern FP method initiated at the time of recruitment for more than 28 days, including stopping FP use altogether or switching to a method other than the four study methods. Additionally, we compared descriptive statistics at baseline for continuers/discontinuers to identify possible differences including characteristics of service delivery, reported reasons for discontinuation, and differences between continuing and discontinuing clients.

## Results

### Client profile

**Figure 1.**
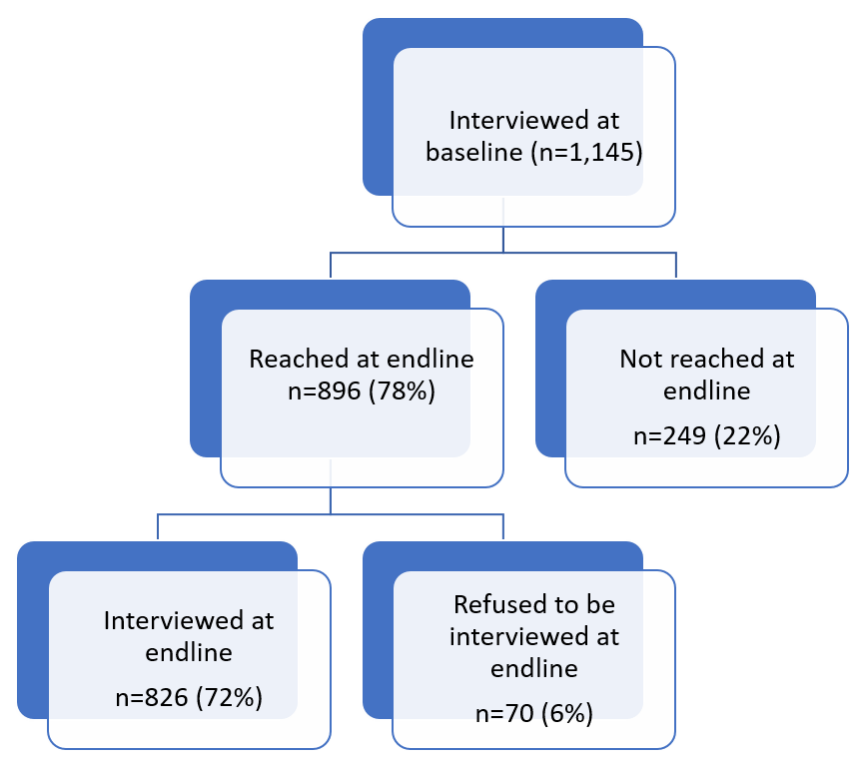
Flowchart of client interviews.

At baseline, 1,145 clients were enrolled through the six strategies. Of those, 72% (826) were reached at endline and agreed to be interviewed; another 6% (70) refused to be re-interviewed. Attrition rates were somewhat higher for outreach strategies as compared with fixed-site, at (25% and 17% respectively). ( Figure 1) We did not observe nonresponse bias based on the method initiated at baseline, nor on the proportion of respondents initiating at fixed-sites versus outreach strategies. The method mix was virtually the same at baseline and at endline; similarly, the proportion of clients who had initiated their method through a fixed-site strategy was nearly the same at 64% in the baseline sample and 66% in the endline sample.

**Figure 2.**
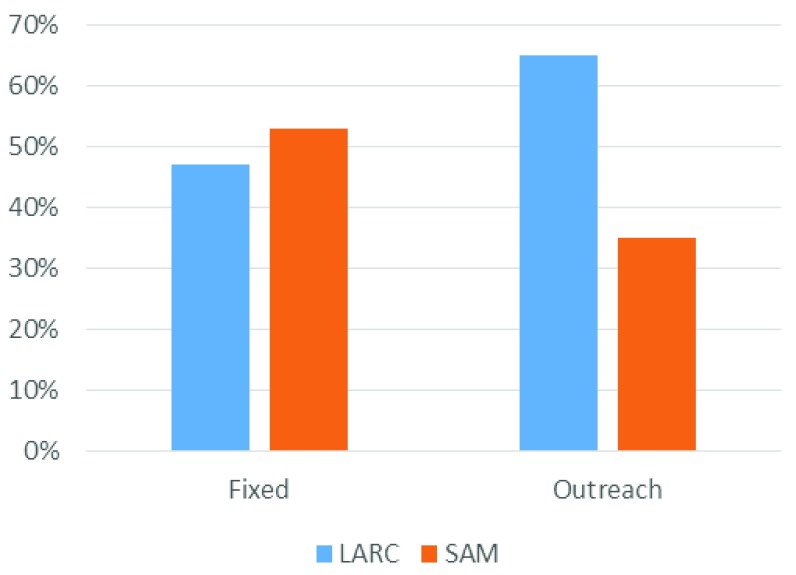
Percent of long acting reversible contraception (LARC) versus short acting methods (SAM) initiated at baseline by type of strategy

Baseline survey results show that the client populations for the fixed-site and outreach models were very similar with regards to age, marriage structure, and religion ( Table 2). One notable difference was that 32% of outreach clients had no formal education, as compared to 23% among fixed-site clients. In addition, outreach clients averaged 3.3 children compared to 2.7 children for fixed-site clients, and 55% of outreach clients had participated in paid work in the past 12 months, as compared to 44% of fixed-site clients. LARC made up a greater proportion of the method mix through outreach strategies than through fixed sites. It made up 65% of methods initiated through the outreach setting and 47% of those initiated through a fixed-site strategy ( Figure 2).

**Table 2.** Demographic information of respondents by strategy.

Demographic Information	Fixed-Site (n=734)	Outreach (n=411)
Average Age	28.2	28.6
% Unmarried	5%	7%
% Partner aware of use of FP	82%	77%
% Muslim	95%	94%
% Used a method prior	49%	49%
% With no education	23%	32%
Average Number of Children	2.7	3.3
% Participated in paid work in last 12 months	44%	55%

FP – Family planning

### Continuation rates

**Figure 3.**
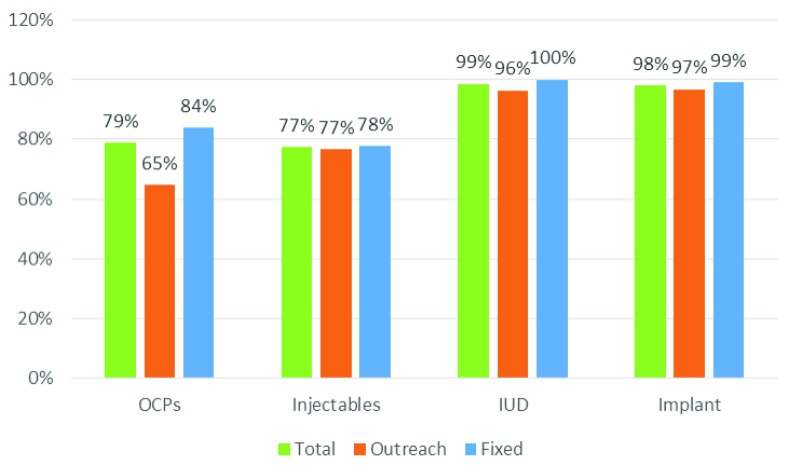
Continuation rates by method initiated and service delivery strategy.

Overall, 88% of the women re-interviewed at endline, approximately seven months after baseline, were still using a modern method of contraception, including 82% who had consistently used the same method, 5% who had switched to one of the other four modern methods, and 2% who had interrupted but re-started the same modern method. Method-specific continuation rates varied from 99% for IUDs to 77% for injectables and were very similar between outreach and fixed-site strategies, with the exception of OCPs. Only 65% of outreach initiators continued using OCPs, compared to 84% of fixed-site initiators. ( Figure 3) One respondent, an implant user who had gotten her method through one of the outreach approaches, reported she had sought to have her device removed, but was denied the service due to lack of access to someone trained on removals.

### Self-reported reasons for discontinuation

Clients who had discontinued or who had switched to a different method were asked to identify the principal reason why; the main responses were experiencing side effects (29%), little or no sexual relations (16%), wanting to get pregnant or had gotten pregnant (14%), and fear of side effects (13%).

### Differences between continuers and discontinuers

The average age of continuing and discontinuing users was similar (28.8 and 28.7 years), but continuing clients had, on average, 3.0 children whereas discontinuers had 2.5 children. Partner awareness of FP use was higher among continuing users (83%) compared to discontinuers (75%). More women were continuing users among those who indicated at baseline that they planned to use their method for more than two years (89%) than among those who indicated at baseline they planned to use for two years or less (70%).

**Figure 4.**
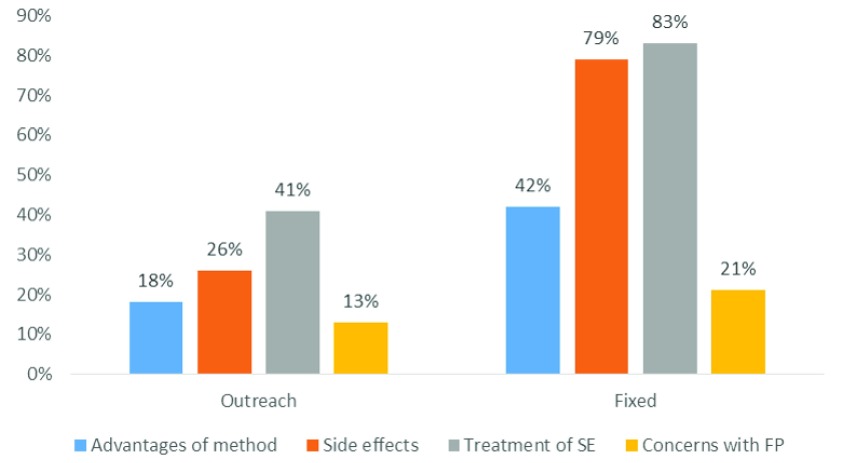
Percent of clients discussing counseling topics with provider.

Across methods, an equal or greater percentage of women who had reported discussing counseling topics with their provider at baseline were continuing users at endline, compared to those who had not discussed previously with their provider. The biggest differences were seen among OCP users and discussions of side effects and injectable users’ discussions of their other concerns with FP (See Table 3). Overall, the proportion of women reporting these counseling topics being covered was smaller at outreach settings than at fixed sites ( Figure 4).

**Table 3.** Continuation rates of respondents who discussed certain topics with their provider.

	Discussed with provider (%)	Did not discuss with provider (%)	Difference (%)
**Advantages of chosen method, other than pregnancy prevention**			
Implants	98.5	97.9	0.6
Injectables	81.5	75.7	5.8
OCPs	81.1	77.4	3.7
**Side effects**			
Implants	98.7	97.1	1.6
Injectables	78.2	75.8	2.4
OCPs	83.1	73.2	9.9
**Treatment/management of side effects**			
Implants	98.1	98.1	0
Injectables	79.8	70.8	9.0
OCPs	83.8	70.2	13.6
**Other concerns with FP**			
Implants	100.0	97.7	2.3
Injectables	85.7	75.9	9.8
OCPs	83.3	77.7	5.6

FP- Family planning, OCP - oral contraceptive pill IUDs not included due to small n’s.

Among the 331 women who had used a method requiring a resupply during the study period (OCPs and injectables), all but one sought these services. Of those, 88% of fixed-site clients sought the refill/reinjection at the same site as their original provision, as compared with 49% of outreach clients. The most common reason clients sought the service at a different location was distance (34%), followed by the source not being available (23%), as would be the case with most of the outreach activities. The most common new source for the refill/reinjection was at a public health post (65%), followed by private sector health facilities (8%) or private sector pharmacies (8%). Of the clients who went to a new source, 92% obtained their method the same day.

## Discussion

Our results indicate that ISSU project strategies allowed most women to continue with their chosen method of FP for the seven-month duration of the study. While not an ideal comparison, seven-month continuation rates achieved through the ISSU project’s FP strategies exceeded 12 month rates reported in Senegal’s 2012–2014 DHS. This was true across all three methods for which DHS data is available. With the exception of OCPs, method specific continuation rates were almost identical between the project’s outreach and fixed-site delivery strategies.

For discontinuing participants, the top two reasons for discontinuation were, respectively, experiencing side effects and little or no sexual relations. These results are consistent with the findings of other studies ^9, 10^. Our analysis showed that in our study population, clients who discussed side effects and how to manage them with the provider, were slightly more likely to continue their use of FP. This suggests that improved counseling could help clients to be better prepared for potential side effects and to manage them better, and thus, continue their use of FP. As noted above, OCP-initiators through outreach strategies had a higher rate of discontinuation as compared to OCP users initiating through a fixed site. Their reasons for discontinuing were varied, however, and most clients in need of resupply found a source, generally through a public facility.

Based on client reports of their discussions with providers on the day of method initiation, the quality of counseling appeared to be higher at fixed sites than through outreach strategies. However, when comparing continuation rates by method between the two types of strategies, they were very similar. Midwives working with the program anecdotally reported that clients who decided to initiate a method through an appointment at a fixed site may have waited to actually obtain and initiate the method until an outreach activity took place nearby, when the method was provided for free, or nearly free. In this case, the client would have already received counseling at the fixed site and may not have repeated it the day she actually initiated the method. When clients were asked in our survey about the counseling they received “that day,” they would not have reported on previous conversations with providers, thus skewing the results for those initiating through an outreach initiative.

As noted above, 90% of clients who intended to use the method for more than two years, including 84% of OCP and injectable users, were still using FP at the time of the endline survey. These results indicate that after seven months, method initiation through the ISSU project interventions is helping most clients to meet their FP needs.

Our study faced a few limitations. It was not within our scope and budget to collect information on catchment areas and draw a random sample of family planning initiators. Thus, we relied on descriptive data which are not generalizable. Due to our study design of follow up survey by telephone, women without a telephone were excluded. It is possible that this excluded the poorest women from the study sample. However, anecdotal information from service providers suggested that most clients have a phone or access to one, and in the end, we only encountered one eligible client without access to a phone. Another limitation was the Senegalese ethics committee disallowance of asking clients the reason for refusing to participate. We ran the risk of non-response bias, but in the end, 98% of eligible women referred to the data collectors were interviewed with success. Lastly, our period of measuring continuation—seven months—was short due to the impending end of the project. The seven-month period, however, did give us enough time that women using OCPs or injectables would need to have found a resupply twice within that timeframe.

## Conclusions

Using both fixed-site and outreach strategies to reach clients with a complete mix of contraceptive methods shows great promise in helping Senegal to increase its CPR. In particular, when offered for free through an outreach setting, a high proportion of clients initiated LARC, and nearly all LARC clients continued that method for at least seven months. Further research into the sensitivity of demand to the price charged is needed, however. For short acting methods, the primary reasons for discontinuation were method specific reasons and not related to finding a source for resupply, demonstrating that outreach efforts are an effective way to reach women selecting short acting methods as well. OCPs were the only method for which there was some difference in continuation rates between outreach and fixed site delivery; better counseling in the outreach setting, where there is limited or no opportunity for follow up, may improve OCP continuation rates.

## Data availability

The data underlying this study is available from Harvard Dataverse.

Dataset 1: Replication Data for: Family planning continuation among the urban poor in Senegal. https://doi.org/10.7910/DVN/UUUZMA ^8^

Data is available under a CC0 1.0 Universal

### Extended data

Base and Endline Surveys used in this study are available from Harvard Dataverse.

Harvard Dataverse: Extended data. Replication Data for: Family planning continuation among the urban poor in Senegal. https://doi.org/10.7910/DVN/UUUZMA ^8^

Data is available under a CC0 1.0 Universal
