# Supplementary material for: To be continued: family planning continuation among the urban poor in Senegal, a prospective, longitudinal descriptive study
Source: Gates Open Res. 2018 Dec 3;2:65. [Version 1] doi: 10.12688/gatesopenres.12880.1 (PMC6362302; doi:10.12688/gatesopenres.12880.1)
Supplement: Peer review history

# Peer review: Reviewer response for version 1

**DOI:** 10.21956/gatesopenres.13972.r26793

**Published:** 2019-01-07

**Authors:** Laura Raney, Alison Bodenheimer Gatto

**Affiliations:** 1 FP2020, UN Foundation (seconded from PHI/Sustaining Technical and Analytical Resources (STAR) project), Washington, DC, USA; 2 Tunjali Group, LLC, Washington, DC, USA; 3 FP2020, UN Foundation, Washington, DC, USA

This research article examines FP continuation rates among the urban poor in Senegal by service delivery mode and method. In this comparison of fixed-site service delivery and community-based outreach service delivery, the findings with regard to discontinuation rates among clients illustrate the importance of the place of outreach for FP. This seven-month study provides evidence that counseling and offer of LARC are feasible in an outreach setting, in addition to the more common offer of counseling and provision of OCPs and injectables. 

 This research article is clearly presented. However, more recent literature is available:  

- 2017 DHS ^1^. Note the current DHS that shows a slight increase in the mCPR for married women in 2017 to 26%. Please consider updating the figures in paragraph one and elsewhere in the text.
- National FP Action Plan 2016 – 2020 ^2^. While the 2012-2015 national plan is what was current at the time of the research, we encourage the authors to include reference to the more recent National Action Plan which was finalized in 2016. Note however, not all the challenges referred to from the previous plan remain challenges in the new plan, i.e., fear of side effects, negative health impacts, and strong social taboos against FP.

In addition, the few articles cited, while seminal and comprehensive, do not cover the full spectrum of the issues. For example,

- There are more recent articles on discontinuation that may be appropriate to consider, e.g. Alkema et al. (2013) ^3^, Sedgh and Hussain (2014) ^4^.
- The study could be strengthened with a further addition on the quality of FP counseling, drawing from the rich literature dating back to Bruce-Jain ^5^ and others (e.g. Harbison (2005) ^6^, and specifically in Senegal: Assaf et al. (2017) ^7^).

 

 The project interventions are presented in a clear manner in Table 1. However, details are missing to further understand, enable replication, and apply lessons learned. (Further details are not available in the questionnaire). While the paper aggregates Fixed-Site and Outreach strategies, the table shows the program strategies are quite different. For example, 

- What methods are midwives allowed to provide? What training do they have? How were the private providers trained? The level of training is important for quality of counsel which can explain differences in continuation rates.
- In the ANSFES Outreach Strategy (Referral by Midwife), the authors might explain how successful the referrals were, which would have an important impact of contraceptive uptake. What percentage of women who were referred to FP services went on to obtain a method at the local service delivery point? Referral is an added step which is where we could lose women. Were referrals a problem?

- It would be useful here to outline task-sharing in Senegal as it pertains to different cadres of providers (i.e., midwives and community health workers) and what methods they are authorized to administer, since it varies so much country to country in Francophone West Africa. In the Enda Santé Mobile Clinic, what level of health worker staffed the mobile clinic and were they authorized to administer all methods themselves without referring?

Figure 2 illustrates the findings that more women chose LARC in outreach strategies (65%) compared to fixed-site strategies (47%). Despite stating that both methods and consultation were offered free of charge in outreach strategies, while clients in fixed-site strategies paid for method and consultation, there is no mention of potential causality.

 Also, from Figure 2, the authors may consider addressing why they think women who received services from fixed sites chose short-acting methods more than women who received services from outreach services. Is quality of counseling received an important factor that may have contributed to this difference in method choice? If so, it might be helpful to take a closer look at the content of counseling. Did the counseling that women received from outreach services include where and when they could access refill/resupply of their chosen method, or other methods if they eventually were to decide to switch? This study seems to underline the importance of quality counseling, and the difference that it can make in continuation rates, however this is one aspect that could be strengthened by adding detail. Perhaps beyond the study scope, but what was the relationship if any between level of health worker, quality of counseling a client received, and rate of continuation? Whether the outreach strategies were conducted primarily by midwives or also by community health workers and other staff has important implications for replicability.

 There is mention (p. 6) but no discussion of the overall issue of accessibility to trained providers for implant and IUD removal, especially in areas where outreach was offered. Do we know if referral was provided for removal when counseling was given, and method was offered/provided?

 The paper concludes that it is feasible to offer LARC and short acting methods through outreach. However, this conclusion is based on the URHI/ISSU project that offered free LARC at outreach. For scale up and longer-term sustainability, the issue of financing looms. The paper does identify the need for further research on willingness to pay and demand. There is a rich history of literature in this field as well as methodology, e.g. Bratt et al.(2005) ^8^, Foreit and Foreit (2003) ^9^, Foreit and Foreit (2005) ^10^.

 Minor:

 The paper refers to modern methods yet cites CPR and not modern CPR (mCPR).

 Table 1 has a typo in last row, middle column – Outreach by MSI where FP services mentioned twice in same sentence.

We have read this submission. We believe that we have an appropriate level of expertise to confirm that it is of an acceptable scientific standard.

## References

[1] Agence Nationale de la Statistique et de la Démographie (ANSD) [Sénégal], et ICF: Sénégal : Enquête Démographique et de Santé Continue (EDS-Continue 2017). *ANSD et ICF*.2018; Reference source

[2] Direction Générale de la Santé, Direction de la Santé de la Reproduction et de la Survie de l’Enfant: Cadre strategique national de planification familiale 2016-2020. *Republique du Senegal Ministère de la Santé et de l’Action Sociale*.2016; Reference source

[3] AlkemaLKantorovaVMenozziCBiddlecomA: National, regional, and global rates and trends in contraceptive prevalence and unmet need for family planning between 1990 and 2015: a systematic and comprehensive analysis. *The Lancet*.2013;381(9878) : 10.1016/S0140-6736(12)62204-1 1642-1652 10.1016/S0140-6736(12)62204-1 23489750

[4] SedghGHussainR: Reasons for contraceptive nonuse among women having unmet need for contraception in developing countries. *Stud Fam Plann*.2014;45(2) : 10.1111/j.1728-4465.2014.00382.x 151-69 10.1111/j.1728-4465.2014.00382.x 24931073

[5] BruceJ: Fundamental Elements of the Quality of Care: A Simple Framework. *Studies in Family Planning*.1990;21(2) : 10.2307/1966669 10.2307/1966669 2191476

[6] HarbisonS: Completing the Demographic Transition in Developing Countries: What does Quality have to do with it?. *IUSSP XXV International Population Conference, Tours, France*.2005; Reference source

[7] AssafSWangWMallickL: Quality of care in family planning services in Senegal and their outcomes. *BMC Health Services Research*.2017;17(1) : 10.1186/s12913-017-2287-z 10.1186/s12913-017-2287-z 28499384PMC5427574

[8] BrattJHAbdel-TawabNIbrahimMAEdreesM: Willingness-to-Pay for Services Provided by the Clinical Services Improvement Project (CSI) in Egypt. *Population Council/Frontiers in Reproductive Health*.2005; Reference source

[9] ForeitJRForeitKG: The reliability and validity of willingness to pay surveys for reproductive health pricing decisions in developing countries. *Health Policy*.2003;63(1) :37-47 1246811610.1016/s0168-8510(02)00039-8

[10] ForeitKGForeitJR: Willingness to Pay Surveys for Setting Prices for Reproductive Health Products and Services: A User's Manual (en Français). *POLICY Project, Futures Group and FRONTIERS, Population Council*.2005; Reference source

# Peer review: Reviewer response for version 1

**DOI:** 10.21956/gatesopenres.13972.r26796

**Published:** 2018-12-20

**Authors:** Julie Solo

**Affiliations:** 1 Independent consultant, Durham, NC, USA

This well-written article provides useful information about the important topic of continuation of FP use.  In particular, with the significant proportion of women receiving FP services from mobile outreach in many countries, it is important to understand what impact this might have on continuation rates of various FP methods.  The study design and results are presented clearly and many of the questions that I had while reading the paper were answered by the end.  There are a few additional pieces of information that would clarify and strengthen the paper to be more helpful in assisting programs.

 Early in the paper, it would be good to add a citation about outreach services to highlight the importance of this mode of service delivery (e.g. High-Impact Practices in Family Planning (HIPs). Mobile outreach services: expanding access to a full range of modern contraceptives. Washington, DC: USAID; 2014 May. Available from: http://www.fphighimpactpractices.org/briefs/mobile-outreach-services). 

 There were multiple program strategies- two categorized as fixed-site and four as outreach.  The paper compares outreach and fixed-site, but were there any notable differences among the different strategies under each category?

 Do you have any data that helps to explain the higher proportion of LARC users among clients reached through outreach?  It would be good to try to explain this, for example by addressing questions around whether there was a difference in the range of methods offered to women at static vs. outreach services or differences in counseling, or whether it is because of the services being free through outreach, etc.  This has been found in other settings (for example see Jarvis et al which found that “clients of mobile outreach and special family planning days preferred LARCs and permanent methods, particularly implants, compared with clients at static services.” The authors speculate that this is mostly due to pre-existing preferences among clients seeking these services as well as the attraction of obtaining usually costly services for free. Jarvis L, Wickstrom J, Shannon C. Client perceptions of quality and choice at static, mobile outreach, and special family planning day services in 3 African countries. Glob Health Sci Pract. 2018;6(3):439-455_

 Page 6 mentions that one implant user was denied removal due to lack of a trained provider- can you explain how this was addressed by the project?

 Later on page 6, the paragraph that begins “Across methods” covers important issues around differences in counseling between continuers and discontinuers- the language could be clarified a bit (for example saying ‘had not discussed previously’ sounds like it could include previous counseling before the counseling session you are asking about).

 It is encouraging to see that women obtaining methods through outreach were able to get resupply from other sources.  Do you have data on whether they were given this information during counseling?  It would be useful for programs to know how to help ensure that this happens. 

 The paper addresses the reason for the seven month follow-up- it would be good to provide a little more detail- do most other studies of continuation look at one-year follow-up?  Are there other studies that have also had shorter follow-up periods?

I have read this submission. I believe that I have an appropriate level of expertise to confirm that it is of an acceptable scientific standard.
